# Evaluation of factors associated with platinum-sensitivity status and survival in limited-stage small cell lung cancer patients treated with chemoradiotherapy

**DOI:** 10.18632/oncotarget.19073

**Published:** 2017-07-07

**Authors:** Qiang Wen, Xue Meng, Peng Xie, Shijiang Wang, Xindong Sun, Jinming Yu

**Affiliations:** ^1^ Department of Radiation Oncology, Shandong Cancer Hospital Affiliated to Shandong University, Shandong University, Jinan, 250117, China; ^2^ Shandong Academy of Medical Sciences, Jinan, 250117, China

**Keywords:** small cell lung cancer, refractory, platinum-sensitivity status, survival, chemoradiotherapy

## Abstract

In this retrospective study, we analyzed the association of clinicopathological factors and therapeutic plans with platinum-sensitivity status and survival of limited-stage small cell lung cancer (LS-SCLC) patients. We enrolled 452 LS-SCLC patients with 279 platinum sensitive and 173 platinum refractory patients. The low serum neuro-specific enolase levels (NSE; *p* = 0.011), neutrophil-to-lymphocyte ratios (NLR; *p* = 0.013) and higher objective response rates (*p* = 0.003) were associated with sensitive group but not the refractory group. Multivariate analysis showed that treatment modality (HR = 0.267, *p* < 0.001), serum lactate dehydrogenase (LDH; HR = 1.894, *p* = 0.016), NLR (HR = 2.043, *p* = 0.043) and platinum-sensitivity status (HR = 0.561, *p* = 0.036) were independent prognostic factors for survival. We further showed that the numbers of chemotherapy cycles and response to first-line therapy were independent prognostic factors for refractory patients only. Our study demonstrates that platinum-sensitivity status is of prognostic importance, as it is strongly associated with survival in LS-SCLC patients.

## INTRODUCTION

Nearly 13%–20% of lung cancer cases are small cell lung cancer (SCLC) [1], which have high incidence of widespread metastasis [2]. About 30% SCLC patients are diagnosed as limited disease (LD) and 70% as extensive disease (ED) [3]. Although patients with limited stage small-cell lung cancer (LS-SCLC) respond well to a combination of chemo- and radio-therapy, high recurrence rates result in low survival [4, 5]. The recurrent cancer is generally refractory to therapy due to drug-resistant cancer cells and remains a problem for LS-SCLC survival [6].

LS-SCLC patients are denoted as platinum refractory or platinum sensitive based on shorter or longer relapse times (90 days from last platinum administered), respectively [7]. Patients with longer treatment-free interval generally demonstrate objective response (OR) to the same chemotherapy regimens used in initial treatment [8–10]. According to the National Comprehensive Cancer Network (NCCN 2016 edition) guidelines, the chosen second-line chemotherapy regimens [11] and the objective response rate (ORR) of second-line treatment were mainly dependent on the length of time from the end of chemotherapy to disease progression [12]. Although platinum-sensitivity status is important in clinical treatments, the risk factors in LS-SCLC are not known.

The circulating tumor cells (CTC) [13] and molecular factors such as vascular endothelial growth factor (VEGF) are widely considered as independent indicators for LS-SCLC survival [14]. However, the association between sensitivity to platinum-based treatment and overall survival is unclear [15]. Therefore, in this retrospective study, we evaluated the correlation of various clinicopathological factors with the platinum-sensitivity status and overall survival of LS-SCLC patients.

## RESULTS

### Patient characteristics

The characteristics of patients were summarized in Table 1. We enrolled 452 LS-SCLC patients with a median follow-up of 35.1 months. Among these, 279 patients were platinum sensitive and 173 patients were platinum refractory. The median age was 56 years (range: 27–82 years) and 75.2% of patients were male. Eastern Cooperative Oncology Group performance status (ECOG PS) score was 0–1 for 357 patients (79%) and 2–3 for 95 patients (21%). 71.2% patients had smoking history and 86.3% of these patients smoked more than 30 pack-years.

**Table 1:** Clinicopathological characteristics

Characteristics	Number of patients	Percentage
**Gender**		
Male	340	75.2%
Female	112	24.8%
**Age**		
< 65	292	64.6%
≥ 65	160	35.4%
**ECOG PS**		
0–1	357	79.0%
2–3	95	21.0%
**Smoking Status**		
Yes	322	71.2%
No	130	28.8%
**Smoking packs**		
≥ 30 packs-year	278	86.3%
< 30 packs-year	44	13.7%
**Treatment modality**		
Chemoradiotherapy	373	82.5%
Chemotherapy alone	79	17.5%
**Chemoradiotherapy**		
Concurrent	303	81.2%
Sequential	70	18.8%
**Treatment regimen**		
Etoposide + platinum	376	83.2%
Irinotecan + platinum	76	16.8%
**Chemotherapy cycles**		
N ≥ 4	396	87.6%
N < 4	56	12.4%
**Therapy response**		
CR + PR	315	69.6%
SD + PD	137	30.3%
**WBC**		
Normal (< 10/nl)	302	70.9%
Elevated (≥ 10/nl)	124	29.1%
**LDH**		
Normal (< 240 U/l)	299	69.1%
Elevated (≥ 240 U/l)	134	30.9%
**Hemoglobin**		
Nomal (≥ 110 g/l)	342	85.7%
Decreased (< 110 g/l)	57	14.3%
**Na**		
Normal (≥ 135 mM)	339	76.5%
Decreased (< 135 mM)	104	23.5%
**GLUT**		
Normal	265	66.4%
Elevated	134	33.6%
**PLT**		
Inside (100–300*10^9^/l)	339	77.9%
Outside (> 300*10^9^/L)	96	22.1%

Abbreviations: ECOG PS: Eastern Cooperative Oncology Group performance status; CR: complete response; PR: partial response; SD: disease stable; PD: disease progression; WBC: white blood count; LDH: lactate dehydrogenase; Na: sodium; GLUT: γ-glutamyl transferase; PLT: platelet count.

In regard to therapeutic variables, 82.5% patients underwent chemoradiotherapy (including concurrent/sequential chemoradiotherapy) and only 17.5% patients underwent chemotherapy alone. All 452 patients underwent platinum-based chemotherapy including 376 (83.2%) with etoposide and 76 (16.8%) with irinotecan. 87.6% patients received at least four cycles of chemotherapy.

### Univariate analysis of clinical factors associated with platinum-sensitivity status

The comparison of clinical variables in the platinum sensitive and refractory patient groups is shown in Table 2. Univariate analysis showed that ECOG PS scores were better in the sensitive patients compared to refractory patients (0–1 in sensitive vs. 0–1 in refractory, 81.7% vs. 74.6%, *p* = 0.070). The objective response (complete plus partial responses) was also higher in the sensitive patients compared to refractory patients (75.3% in sensitive vs. 60.7% in refractory, *p* = 0.001). Furthermore, more number of sensitive patients received chemoradiotherapy compared to the refractory group (85.3% vs. 78.0% *p* = 0.048). The tumor size (*p* = 0.149) and white blood counts (WBC; *p* = 0.306) were statistically similar between the two groups of patients. The neuro-specific enolase (NSE) levels were lower in the sensitive patients compared to refractory patients (44.44 vs. 52.26 at diagnosis, *p* = 0.004; 17.96 vs. 23.16 after four cycles of chemotherapy, *p* < 0.001; and 50.27 vs. 62.45 at progression, *p* < 0.001). Also, the neutrophil-to-lymphocyte ratios (NLR) were lower in the sensitive group compared to the refractory group (3.83 vs. 4.66, *p* = 0.003 at diagnosis; 2.45 vs. 4.1 after four cycles of chemotherapy, *p* < 0.001; 4.12 vs. 5.11, *p* = 0.003 at progression).

**Table 2:** Univariate analysis of relation between clinicopathological factors and platinum-sensitivity status in LS-SCLC

Characteristics	Sensitive	Refractory	*p*
**Gender**			
Male	213 (76.3%)	127 (73.4%)	0.483
Female	66 (23.7%)	46 (26.6%)	
**Age**			
< 65	187 (67.0%)	105 (60.7%)	0.171
≥ 65	92 (33.0%)	68 (39.3%)	
**ECOG PS**			
0–1	228 (81.7%)	129 (74.6%)	0.07
2–3	51 (18.3%)	44 (25.4%)	
**Smoking Status**			
Yes	192 (68.8%)	130 (75.1%)	0.149
No	87 (31.2%)	43 (24.9%)	
**Smoking packs**			
≥ 30 packs-year	128 (87.1%)	150 (85.7%)	0.723
< 30 packs-year	19 (12.9%)	25 (14.2%)	
**Treatment modality**			
Chemoradiotherapy	238 (85.3%)	135 (78.0%)	0.048
Chemotherapy alone	41 (14.7%)	38 (22.0%)	
**Chemoradiotherapy**			
Concurrent	224 (83.3%)	79 (76.0%)	0.179
Sequential	45 (16.7%)	25 (24.0%)	
**Therapy regimen**			
Etoposide + platinum	235 (84.2%)	141 (81.5%)	0.451
Irinotecan + platinum	44 (15.8%)	32 (18.5%)	
**Chemotherapy cycles**			
*N* ≥ 4	250 (89.6%)	146 (84.4%)	0.102
*N* < 4	29 (10.4%)	27 (15.6%)	
**Response**			
CR + PR	210 (75.3%)	105 (60.7%)	0.001
SD + PD	69 (24.7%)	68 (39.3%)	
**WBC**			
Normal (< 10/nl)	189 (72.7%)	113 (68.1%)	0.306
Elevated (≥ 10/nl)	71 (27.3%)	53 (31.9%)	
**LDH**			
Normal (< 240 U/l)	193 (71.5%)	106 (65.0%)	0.159
Elevated (≥ 240 U/l)	77 (28.5%)	57 (35.0%)	
**Hemoglobin**			
Normal (≥ 110 g/l)	211 (86.8%)	131 (84.0%)	0.426
Decreased (< 110 g/l)	32 (13.2%)	25 (16.0%)	
**Na**			
Normal (≥ 135 mM)	208 (75.9%)	131 (82.4%)	0.699
Decreased (< 135 mM)	66 (24.1%)	28 (17.6%)	
**GLUT**			
Normal (≤ 45 U/l)	158 (65.0%)	107 (68.6%)	0.461
Elevated (> 45 U/l)	85 (35.0%)	49 (31.4%)	
**PLT**			
Inside (100–300*10^9^/l)	208 (78.8%)	131 (76.6%)	0.592
Outside (< 100 or > 300*10^9^/l)	56 (21.2%)	40 (23.4%)	
**Tumor Size**	50.78 ± 20.58	53.81 ± 21.67	0.149
**BMI**	23.56 ± 2.84	23.13 ± 2.89	0.119
**CEA**			
At diagnosis	9.80 ± 3.38	10.38 ± 3.89	0.11
After four cycles	8.09 ± 2.75	8.52 ± 2.51	0.184
At progression	8.38 ± 3.35	8.94 ± 3.06	0.276
**NSE**			
At diagnosis	44.44 ± 23.28	52.26 ± 25.58	0.004
After four cycles	17.96 ± 7.54	23.16 ± 10.71	< 0.001
At progression	50.27 ± 26.99	62.45 ± 33.60	< 0.001
**Cyfra21-1**			
At diagnosis	2.67 ± 2.43	3.07 ± 2.37	0.109
After four cycles	1.86 ± 1.34	2.09 ± 1.44	0.215
At progression	2.15 ± 1.70	2.41 ± 1.56	0.186
**NLR**			
At diagnosis	3.83 ± 2.31	4.66 ± 2.82	0.003
After four cycles	2.45 ± 1.67	4.1 ± 2.56	< 0.001
At progression	4.12 ± 2.49	5.11 ± 3.09	0.003
**PLR**			
At diagnosis	178.68 ± 95.34	186.18 ± 109.55	0.462
After four cycles	170 ± 95.31	179.55 ± 105.55	0.362
At progression	176.77 ± 99.6	184.83 ± 98.89	0.395

Abbreviations: LS-SCLC: limited-stage small cell lung cancer; ECOG PS: Eastern Cooperative Oncology Group performance status; CR: complete response; PR: partial response; SD: disease stable; PD: disease progression; WBC: white blood count; LDH: lactate dehydrogenase; Na: sodium; GLUT: γ-glutamyl transferase PLT: platelet count; BMI: body mass index; CEA: carcino-embryonic antigen; NSE: neuro-specific enolase; NLR: neutrophil-to-lymphocyte ratio; PLR: platelet-to-lymphocyte ratio.

### Multivariate analysis of clinical factors associated with platinum-sensitivity status

Multivariate analysis demonstrated that among all the factors analyzed, only NSE, NLR and objective response correlated with the platinum-sensitivity status (Table 3). Objective response was an independent factor associated with platinum sensitive patients (OR = 0.375, 95% CI: [0.195, 0.722]; *p* = 0.003). Meanwhile, NSE levels and NLR ratios at diagnosis (*p* = 0.011, *p* = 0.013), after four cycles of chemotherapy (*p* < 0.001, *p* = 0.002) and at the time of progression (*p* < 0.001, *p* = 0.030) were independent factors associated with platinum-sensitivity status. The other clinical parameters including ECOG PS (OR = 2.221, 95% CI [0.681, 7.249]; *p* = 0.186) and treatment modality (OR = 0.647, 95% CI [0.215, 1.953]; *p* = 0.440) were not associated with platinum sensitivity (Table 3).

**Table 3:** Multivariate analysis of relation between clinicopathological factors and platinum-sensitivity status in LS-SCLC

Characteristics	OR	95 CI%	*P*
**ECOG PS (PS 2–3)**	2.221	0.681–7.249	0.186
**Treatment modality** (Chemoradiotherapy)	0.647	0.215–1.953	0.440
**Response (CR + PR)**	0.375	0.195–0.722	0.003
**NSE**			
At diagnosis	1.243	1.062–1.484	0.011
After four cycles	2.726	2.035–3.684	< 0.001
At progression	1.812	1.454–2.272	< 0.001
**NLR**			
At diagnosis	1.703	1.054–2.757	0.013
After four cycles	2.130	1.721–2.656	0.002
At progression	1.743	1.056–2.747	0.030

Abbreviations: LS-SCLC: limited-stage small cell lung cancer; OR: Odds Ratio; CI: confidence interval; ECOG PS: Eastern Cooperative Oncology Group performance status; CR: complete response; PR: partial response; NSE: neuro-specific enolase; NLR: neutrophil-to-lymphocyte.

We then compared mean NLR and platelet-to-lymphocyte ratio (PLR) to disease status (Table 4). In platinum-sensitive group, NLR decreased from 3.83 to 2.45 (*p* < 0.001) after four cycles of chemotherapy, and then increased to 4.12 (*p* < 0.001) at progression. In the refractory group, NLR decreased from 4.66 to 4.10 (*p* = 0.03), but sharply increased to 5.11 at progression (*p* = 0.006). PLR also demonstrated similar trend in the two patient groups, although the differences were not significant when compared in terms of disease status (Table 4).

**Table 4:** Comparison of NLR and PLR at diagnosis, after four cycles of chemotherapy and at disease progression

Factors	At diagnosis	After 4 cycles	*p*	After 4 cycles	Progression	*p*
**Sensitive**						
NLR	3.83 ± 2.31	2.45 ± 1.67	< 0.001	2.45 ± 1.67	4.12 ± 2.49	< 0.001
PLR	178.68 ± 95.34	170 ± 95.31	0.335	170 ± 95.31	176.77 ± 99.6	0.52
**Refractory**						
NLR	4.66 ± 2.82	4.1 ± 2.56	0.03	4.1 ± 2.56	5.11 ± 3.09	0.006
PLR	186.18 ± 109.55	179.55 ± 105.55	0.601	179.55 ± 105.55	184.83 ± 98.89	0.749

Abbreviations: NLR: neutrophil-to-lymphocyte ratio; PLR: platelet-to-lymphocyte ratio.

### Prognostic factors for LS-SCLC survival

**Figure 1:**
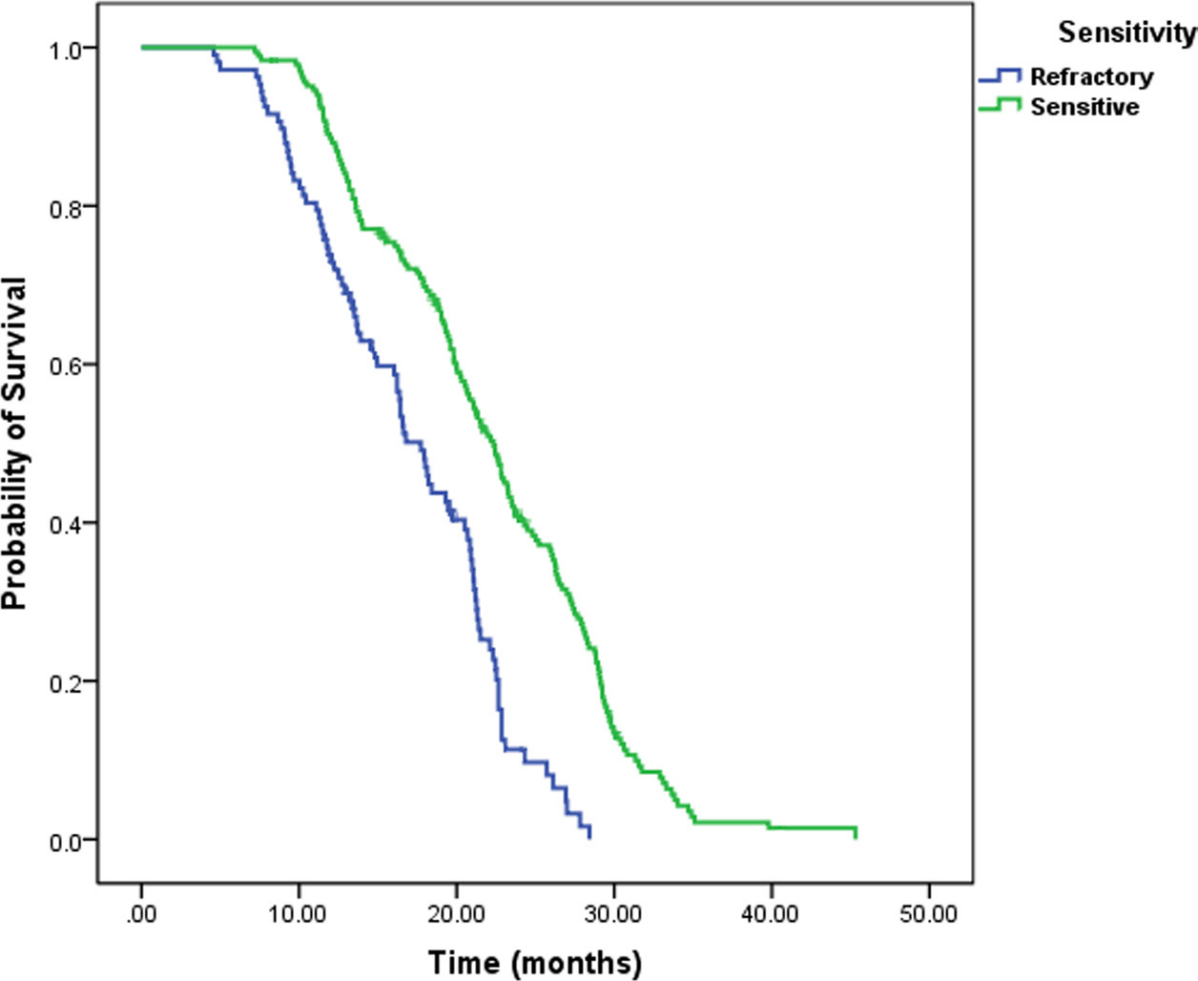
Kaplan–Meier analysis of OS for platinum-sensitivity status Patients with platinum sensitive achieved longer OS than patients with platinum refractory.

**Figure 2:**
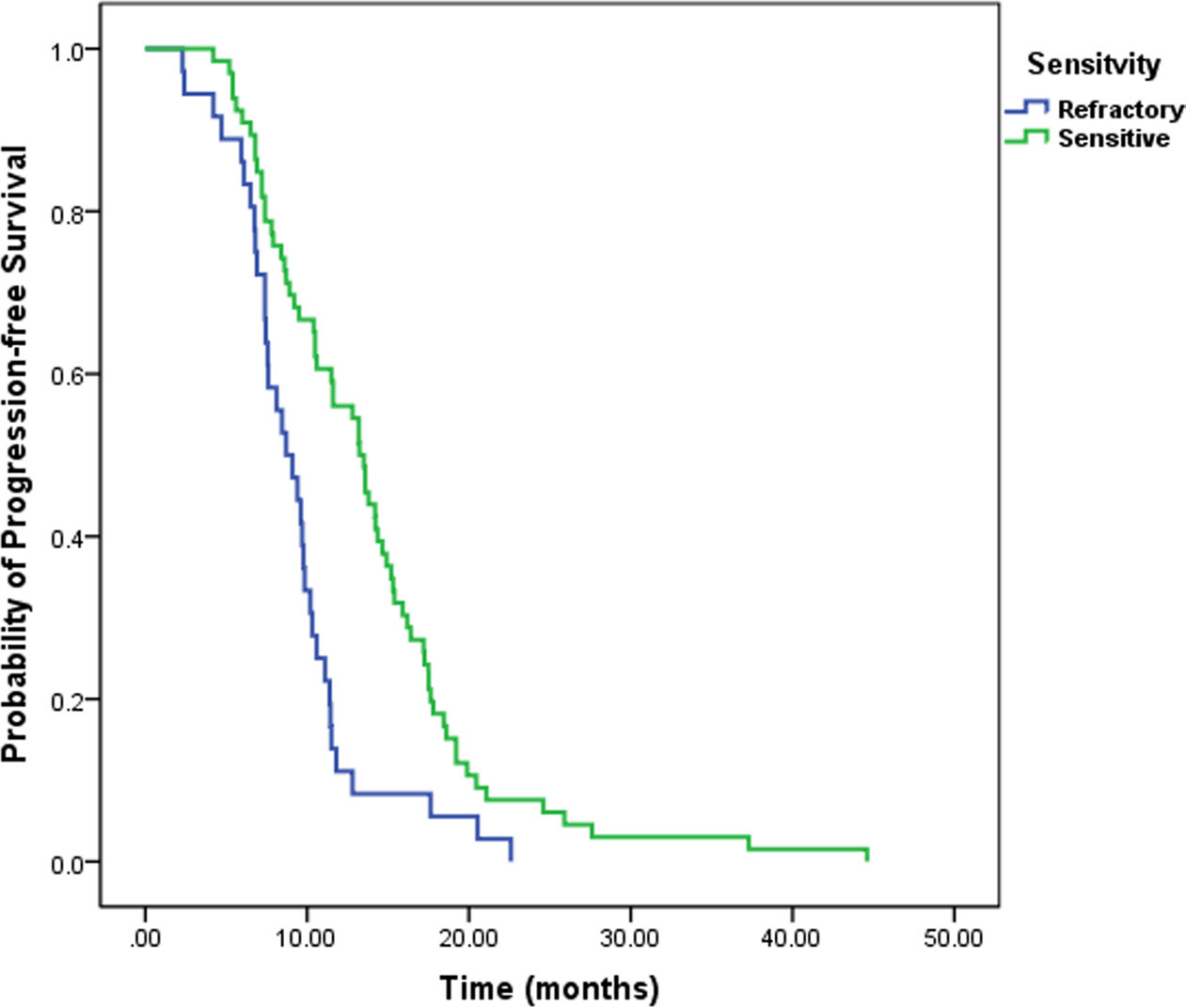
Kaplan–Meier analysis of PFS for platinum-sensitivity status Patients with platinum sensitive had significant better PFS than patients with platinum refractory.

The median overall survival (OS) of LS-SCLC patients was 19.7 months and median progression-free survival (PFS) was 10.4 months. Platinum-sensitivity status affected survival and PFS rates differentially. The platinum sensitive group demonstrated better survival than the refractory group (21.1 months vs.16.8 months, log rank *p* < 0.001, Figure 1). The PFS rates were also better for the sensitive group in comparison to the refractory patients (13.2 months vs. 8.4 months, log rank *p* < 0.001, Figure 2).

Univariate analysis showed that treatment modality (*p* < 0.001), number of chemoradiotherapy cycles (*p* = 0.005), lactate dehydrogenase (LDH) (*p* = 0.001), platinum-sensitivity status (*p* < 0.001), NSE (*p* = 0.065), NLR (*p* = 0.004) and PLR (*p* = 0.016) were associated with survival. Multivariate analysis revealed that patients that received combined treatment showed increased OS compared to chemotherapy alone (HR = 0.267, 95% CI [0.128, 0.555]; *p* < 0.001). Furthermore, high serum LDH levels (HR = 1.894, 95% CI [1.124, 3.193]; *p* = 0.016) and NLR levels (HR = 2.043, 95% CI [1.017–4.098]; *p* = 0.043) were independent negative prognostic factors of survival. The platinum sensitive patients correlated with better overall survival (HR = 0.561 95% CI [0.327–0.962], *p* = 0.036; Table 5).

**Table 5:** Multivariate analysis of prognostic factors associated with survival and PFS

Characteristics	MST	HR (95% CI)	*p*	PFS	HR (95% CI)	*p*
**Numbers of cycle**						
*N* ≥ 4	20.5	0.753	0.448	11.4	0.271	< 0.001
		0.362–1.566			0.141–0.521	
*N* < 4	17.0	Ref.		6.5	Ref.	
**Treatment Modality**						
Chemoradiotherapy	21.5	0.267	< 0.001	11.6	0.349	< 0.001
		0.128–0.555			0.176–0.615	
Chemotherapy	15.3	Ref.		7.0	Ref.	
**LDH**						
Elevated (> 240 U/l)	17.5	1.894	0.016	7.5	1.952	0.008
		1.124–3.193			1.195–3.188	
Normal (≤ 240 U/l)	22.6	Ref.		13.5	Ref.	
**NSE**						
Elevated (> 18 ng/ml)	18.1	1.373	0.117	9.1	1.513	0.020
		0.852–2.231			1.292–1.902	
Normal (≤ 18 ng/ml)	22.8	Ref.		14.5	Ref.	
**NLR**						
Elevated (≥ 4)	17.8	2.043	0.043	8.2	1.748	0.016
		1.017–4.098			1.110–2.753	
Normal (< 4)	23.2	Ref.		13.8	Ref.	
**Platinum Status**						
Sensitive	21.1	0.561	0.036	13.2	0.333	< 0.001
		0.327–0.962			0.197–0.565	
Refractory	16.8	Ref.		8.4	Ref.	
**Response**						
CR + PR	21.8	0.592	0.076	13.0	0.384	0.021
		0.333–1.054			0.187–0.808	
SD + PD	17.0	Ref.		7.8	Ref.	

Abbreviations: PFS: progression-free time; MST: median survival time; HR: hazard ratio; CI: confidence interval; LDH: lactate dehydrogenase; NSE: neuro-specific enolase; NLR: neutrophil-to-lymphocyte ratio; CR: complete response; PR: partial response; SD: disease stable; PD: disease progression.

Similarly, patients with high serum LDH (*p* = 0.008), NSE (*p* = 0.02) and NLR (*p* = 0.016) demonstrated decreased PFS rate. Among treatment-related variables, patients with chemoradiotherapy showed better PFS than chemotherapy alone (11.6 months vs. 7.0 months, *p* < 0.001). Also, no less than four cycles of chemotherapy (*p* < 0.001), platinum sensitive group (*p* < 0.001) and best response to initial chemotherapy (*p* = 0.021) were all favorable factors for PFS (Table 5).

### Subgroup analysis of clinical factors associated with survival

In subgroup analysis, numbers of chemotherapy cycles (HR = 0.515, 95% CI [0.306, 0.865], *p* = 0.012), objective response (HR = 0.683, 95% CI [0.512–0.906], *p* = 0.011) were independent prognostic factors for refractory patients, but not for sensitive patients (Table 6). Elevated NLR and NSE level, less than four cycles of chemotherapy, chemotherapy alone and resistance to first-line treatment in both groups demonstrated poor PFS. LDH levels did not correlate with PFS in refractory LS-SCLC patients (*p* = 0.382; Table 7).

**Table 6:** Multivariate analysis of prognostic factors associated with survival in sensitive and refractory patients

Variables	Sensitive LS-SCLC			Refractory LS-SCLC		
	MST	HR (95% CI)	*p*	MST	HR (95% CI)	*p*
**Numbers of cycle**						
*N* ≥ 4	23.8	0.598 0.345–1.034	0.066	18.3	0.515 0.306–0.865	0.012
*N* < 4	17.5	Ref.		13.1	Ref.	
**Response**						
CR + PR	24.5	0.571 0.309–1.068	0.083	18.4	0.683 0.512–0.906	0.011
PD + SD	17.1	Ref.		14.2	Ref.	
**Treatment Modality**						
Chemoradiotherapy	24.7	0.528 0.315–0.878	0.016	18.0	0.738 0.552–0.943	0.023
Chemotherapy	16.6	Ref.		14.6	Ref.	
**LDH**						
Elevated (> 240 U/l)	18.1	2.610 1.862–3.679	< 0.001	13.7	2.313 1.556–3.417	< 0.001
Normal (≤ 240 U/l)	26.4	Ref.		19.2	Ref.	
**NLR**						
Elevated (≥ 4)	18.4	1.538 1.072–2.214	0.024	14.1	1.677 1.110–2.524	0.013
Normal (< 4)	25.6	Ref.		18.6	Ref.	

Abbreviations: LS-SCLC: limited-stage small cell lung cancer; MST: median survival time; HR: hazard ratio; CI: confidence interval; CR: complete response; PR: partial response; SD: disease stable; PD: disease progression; LDH: lactate dehydrogenase; NLR: neutrophil-to-lymphocyte ratio.

**Table 7:** Multivariate analysis of prognostic factors associated with PFS in sensitive and refractory patients

Variables	Sensitive LS-SCLC			Refractory LS-SCLC		
	PFS	HR (95% CI)	*p*	PFS	HR (95% CI)	*p*
**Numbers of cycle**						
*N* ≥ 4	14.0	0.329 0.119–0.906	0.031	10.4	0.289 0.117–0.716	< 0.001
*N* < 4	8.3	Ref.		5.9	Ref.	
**Treatment Modality**						
Chemoradiotherapy	14.6	0.283 0.106–0.755	0.012	9.1	0.316 0.102–0.960	0.042
Chemotherapy	7.8	Ref.		6.2	Ref.	
**LDH**						
Elevated (> 240 U/l)	8.5	2.331 1.211–4.486	0.001	7.0	1.366 0.678–2.753	0.382
Normal (≤ 240 U/l)	15.3	Ref.		9.0	Ref.	
**NLR**						
Elevated (≥ 4)	9.2	1.748 1.110–2.753	0.016	6.7	1.283 1.088–1.546	0.037
Normal (< 4)	15.4	Ref.		9.4	Ref.	
**NSE**						
Elevated (> 18 ng/ml)	10.2	1.513 1.292–1.902	0.020	6.0	1.723 1.278–2.127	< 0.001
Normal (≤ 18 ng/ml)	16.0	Ref.		10.0	Ref.	
**Response**						
CR + PR	15.0	0.266 0.116–0.614	0.002	9.3	0.342 0.163–0.718	0.006
SD + PD	8.7	Ref.		6.0	Ref.	

Abbreviations: LS-SCLC: limited-stage small cell lung cancer; PFS: progression-free time; HR: hazard ratio; CI: confidence interval; LDH: lactate dehydrogenase; NLR: neutrophil-to-lymphocyte ratio; NSE: neuro-specific enolase; CR: complete response; PR: partial response; SD: disease stable; PD: disease progression.

## DISCUSSION

In this retrospective study of LS-SCLC patients, we evaluated the correlation between clinical test results and factors with platinum-sensitivity status and survival. Our analysis revealed that NSE, NLR and objective response were independent predictors of platinum-sensitivity status. Prognostic factors for survival included treatment modality, LDH, NLR and platinum-sensitivity status.

While the first-line treatment guidelines are clear, there is ambiguity regarding treatment for relapsed LS-SCLC patients. Ardizzoni *et al*. demonstrated that response to the second-line drug, topotecan, was 38% and 10% in sensitive and refractory patients, respectively [16]. In the latest meta-analysis, Horita *et al*. demonstrated that topotecan response rates for sensitive and refractive patients were 17% and 5% and the corresponding one-year OS rates were 27% and 9%, respectively [17]. Therefore, distinguishing sensitive and refractory LS-SCLC patients early could result in therapeutic benefits.

NSE levels are associated with response to therapy, disease stage and overall survival and it can be used to monitor disease progression and recurrence. Yan *et al* demonstrated that survival rates were significantly different between low and high NSE level groups in small cell carcinoma of esophagus [18]. The histological characteristic of small cell carcinoma of esophagus was similar to SCLC. Therefore, our data showed that the sensitivity of platinum-based chemo-radiotherapy was associated with serum NSE levels before treatment.

Recently, a series of inflammatory factors, such as NLR, PLR and C-reactive protein (CRP) were shown to be correlated with a poor prognosis in various types of cancer [19–21]. NLR was involved with tumor growth, invasion and metastasis through neutrophil elastase activity and suppression of the adaptive immune system [22, 23]. However, NLR was not a stable tumor-specific biomarker since it was easily affected by treatment and radiation-induced inflammation [24, 25]. Therefore, some reports suggested that other inflammatory factors should be evaluated with NLR [19, 26]. Furthermore, NLR levels always changed with treatment and disease courses [27]. Our results suggested that NLR was a critical parameter in evaluating tumor response and monitoring disease progression in LS-SCLC.

We also evaluated if therapeutic efficacy could distinguish sensitive and refractory patients. Nagy-Mignotte *et al*. demonstrated complete response in 60.1% sensitive patient group compared to 9.7% in the refractory patient category [12]. Johnson *et al*. demonstrated effective response in 12.5% sensitive SCLC patients [28]. Therefore, NSE, NLR and objective response provide reliable and important predictive information about platinum-sensitivity status that would be helpful to weigh benefits of different treatments in the two groups.

Many studies have reported that good PS score, younger age and early stage are associated with survival benefit in LS-SCLC [29–31]. However, there is ambiguity due to variable biomarkers usage and analysis limitations. Some studies have suggested that radiotherapy in LS-SCLC patients yields a 5–10% improvement in two-year survival rate, and a corresponding 20%–35% increase in local control compared to chemotherapy alone [32, 33]. Our results indicated that patients with combined therapy were associated with decreased hazard ratios (HRs) compared to chemotherapy alone. Furthermore, many studies also suggested that early concurrent thoracic radiation was superior to late concurrent and sequential radiotherapy [34–36]. There was no association between sequential thoracic therapy and survival in our study, because it was more common in younger patients or patients with good condition (PS < 2). Thirdly, radiation schedule and dose were still worthy to deeply analyze. Turrisi *et al*. concluded that patients that received radiotherapy twice-daily in 3 weeks showed better survival than those that received radiotherapy once-daily in 5 weeks (MST, 23 months vs. 19 months, *p* = 0.04) [37]. High-dose radiotherapy (60 Gy) showed better survival and response rates compared to low-dose radiation therapy in the once-daily treatments [38–40].

Bremnes *et al*. concluded that LDH was an effective prognostic predictor in 219 patients with limited disease [41]. Moreover, Stokkel reported that LDH was an independent prognostic factor for tumor progression and patient survival [42]. However, Li *et al*. showed that high LDH levels were more frequent in advanced-stage SCLC patients and did not correlate with long-term survival [43]. Similarly, Brueckl *et al*. demonstrated that LDH was not associated with OS due to its high correlation with WBC [44]. The differences among the results in these studies might be due to different approaches to measuring survival, disparate study designs or variables and sample sizes. Establishing an association between serum LDH level and SCLC progression would be beneficial to effectively monitoring therapy response, as previously shown [45, 46].

The prognostic role of NLR in LS-SCLC has not been investigated in detail. Kang *et al*. demonstrated that patients with low NLR (NLR < 4) at diagnosis had relatively longer OS and PFS compared to those with high NLR (≥ 4) [27]. However, Wang *et al*. did not find significant association between NLR and OS in LS-SCLC [47]. Apart from assessing the prognostic role of NLR, the cut-off value of NLR is worth to be discussed. Some studies demonstrated significant correlation between NLR and overall survival with different cutoff values of NLR [20, 21]. Yamanaka *et al*. showed that advanced gastric cancer patients with low NLR (< 2.5) had better OS [48].

We observed a clear cut survival benefit for platinum sensitive LS-SCLC patients. Von Pawel *et al*. reported that the sensitive group among the 637 SCLC patients that received topotecan or amrubicin had better median survival (Topotecan: 10 months vs. 5.7 months; Amrubicin: 9.2 months vs. 6.2 months) [49]. Garassion *et al*. considered that platinum-sensitivity status had significant effects on ORR (34.5% vs. 17.5%, *p* = 0.06) and OS (9.2 months vs. 5.8 months, *p* = 0.08) of LS-SCLC patients [50]. However, there are some contradictory reports. Lara *et al*. investigated 3 clinical trials based on advanced stage SCLC and did not find association between platinum-sensitivity status and progression-free survival (PFS) (*p* = 0.49) or overall survival (OS) (*p* = 0.14) [51]. This study used different methodologies for clinical staging and data collection compared to our study. Based on our data, we postulate that response to platinum-based chemotherapy is prognostic.

Adequate chemotherapy doses can significantly improve the short-term benefits and PFS of LS-SCLC patients. In the present study, we observed that patients treated with no less than four cycles of chemotherapy showed higher median survival although the data was not statistically significant in multivariate analysis. This could be because most of patients cannot tolerate six cycles of chemotherapy, especially concurrent chemoradiotherapy due to serious side effects like gastrointestinal irritation and bone marrow suppression. Furthermore, long term chemotherapy before radiotherapy led to drug-resistant cancer cells that formed small distance metastases and reduced the efficiency of radiotherapy [52].

Subgroup analysis demonstrated that more than four cycles of chemotherapy and objective response to initial treatment were favorable prognostic factors only in refractory patients. This was probably due to different patient characteristics and response to successive lines of chemotherapy in two groups. The refractory group was mostly made up of elderly and poor PS patients, with greater reliance on chemotherapy cycle numbers and the drug dose. Nagy-Mignotte *et al*. showed that sensitive patients benefit more than refractory patients from subsequent therapy [12]. As a consequence, the response to initial therapy was critical in improving disease prognosis.

Serum LDH levels were related with PFS in the sensitive group, but not in the refractory group. Previous studies demonstrated that elevated LDH levels were associated with poor survival and chemo-/radio resistance in malignancies as they signified hypoxia inducible factor induced tumor aggressiveness [53].

Apart from basic clinical and laboratory factors, many molecular and genetic studies have explored novel prognostic factors that require invasive examinations and increase the economic burden on patients [54, 55]. Our study suggested that routine laboratory test results are prognostic and can provide information for precision treatment. By identifying patients with sensitive disease, patients can be treated with lower total radiotherapy and chemotherapy doses to achieve good results. At the same time, patients that are more likely to progress can be switched to alternative treatments earlier to reduce high drug toxicity.

This study is limited because it is retrospective and due to heterogeneity of the characteristics and therapies administered in individual trials. Furthermore, confounding factors like race and socioeconomic status [56, 57], which were not taken into consideration in this study. Also, the small number of patients and the single center study can result in intrinsic bias. Finally, since the radiotherapy technique is undergoing constant improvements, the platinum-sensitivity status between patients at different times is hard to compare in a retrospective study. Therefore, multi-center prospective study with larger patient pools is needed to confirm our results. In conclusion, our study demonstrates that platinum-sensitivity status is a critical prognostic factor that determines the survival rates in LS-SCLC patients.

## MATERIALS AND METHODS

### Patient enrollment criteria

We retrospectively enrolled LS-SCLC patients that were diagnosed by cytology or histology between January 2005 and December 2010 at Shandong Cancer Hospital and received at least one cycle of chemotherapy. The cytological or histological diagnosis of disease was performed by mediastinoscopy, bronchofiberoscopy and biopsy of lymph nodes. For all patients, standard evaluation before treatment included brain magnetic resonance imaging (MR), bone scintigraphy and CT imaging of chest and abdomen. Positron emission tomography-computed tomography (PET/CT) was not routinely performed. Tumor stage was determined according to the Veterans Administration Lung Study Group system [58]. This study was approved by the Research Ethics Committee of Shandong Cancer Hospital, China. Informed consent was obtained from all participants.

### Therapeutic treatment and tumor response

Patients that did not receive standard therapeutic strategy were excluded from this study. The chemotherapy regimens included etoposide plus platinum and irinotecan plus platinum. Chemotherapy doses were modified based on individual toxicity levels and blood counts. Patients underwent radiotherapy either by 3-dimensional conformal radiotherapy (3D-CRT) or intensity modulated radiotherapy (IMRT). The gross tumor volume (GTV) included primary tumor and positive lymph nodes. The clinical tumor volume (CTV) was drawn from GTV with 8 mm margin. The planning target volume (PTV) was determined from GTV with less than 1.5 cm in 3D.

Patients with disease progression in less than 90 days after treatment were denoted as platinum refractory, whereas patients with disease progression at 90 or more days were identified as platinum sensitive. During chemotherapy, CT scan and the specific biomarkers of tumor were accessed and analyzed every two or three cycles to determine tumor response. Based on Response Evaluation Criteria in Solid Tumor (RECST) guidelines [59], tumor response to first-line treatment was sub-divided into complete response (CR), partial response (PR), stable disease (SD) and progression disease (PD).

### Follow-up and data collection

The clinical data was obtained from medical records. Re-examinations included physical examination, tumor biomarkers, routine laboratory test, medication history and CT scan. Chest and abdomen CT imaging was performed one month after initial treatment, with follow-up every 2–3 months in the first year, then every sixth months, second year onwards. Brain MR and bone scintigraphy were not compulsory and were administered according to clinical requirements.

In this study, clinical and demographic indicators including age, gender, smoking status, ECOG PS and treatment modality were collected using medical record system at baseline. Also, routine laboratory tests that were obtained from patient records included WBC, hemoglobin (HB), PLT, LDH, sodium (Na), γ-glutamyl transferase (GLUT) at diagnose. Cyfra21-1, NSE, carcinoembryonic antigen (CEA), NLR and PLR were recorded at diagnosis, after four cycles of chemotherapy, and at the time of progression. The NLR was calculated from the differential counts by dividing the neutrophil number by the lymphocyte number. The PLR was calculated by dividing the platelet count by the lymphocyte count. The cutoff values of variables were determined using the clinical normal range and the related research results [27]. Parameters with more than 20% missing observations were excluded from the study.

### Statistical analysis

Survival time was measured from the time of diagnosis to death or the last follow-up date. Progression-free survival was determined from the time of therapy initiation to the time of disease progression or death. Date of recurrence was determined by the date of positive CT imaging results. The platinum-sensitivity status was determined based on the time between last platinum administrations to date of recurrence.

All statistical analyses were performed using SPSS software version 19.0 for windows (SPSS Inc., Chicago, IL, USA). Categorical variables were expressed as percentage and compared between groups using chi-square test. Continuous variables were presented as mean ± standard deviation (s.d.) or median and range with a Mann-Whitey *U* test performed for comparison. Variables with *p* < 0.1 were considered statistically significant (two-sided). Logistic regression with backward stepwise method was used for multivariate analysis. Patient survival curves were measured by the Kaplan-Meier method and compared by log-rank test. Univariate Cox proportional hazard models were used to identify factors associated with survival and PFS. Multivariate Cox regression analyses were performed to determine the factors that were significant based on univariate analysis. Variables with *p* < 0.05 were considered statistically significant (two-sided).
